# Micro‐Galvanic Coupling Programs the Therapeutic Zinc Ion Window to Reconfigure Immune Cascades for Pro‐Regenerative Bone Healing

**DOI:** 10.1002/advs.76084

**Published:** 2026-06-12

**Authors:** Chaoyang Sun, Bo Jia, Jiahui Shi, Shuang Li, Guo Bao, Dong Bian, Kai Chen, Junlong Tan, Xuenan Gu, Yan Guan, Yu Qin, Xinhua Qu, Xiaogang Wang, Yufeng Zheng, Hongtao Yang

**Affiliations:** ^1^ School of Engineering Medicine Beihang University Beijing China; ^2^ Department of Joint Replacement, Sports Medicine, and Trauma,Department of Orthopedics, Renji Hospital Shanghai Jiaotong University School of Medicine Shanghai China; ^3^ School of Materials Science and Engineering Peking University Beijing China; ^4^ Department of Reproduction and Physiology National Research Institute For Family Planning Beijing China; ^5^ Medical Research Institute, Department of Orthopedics, Guangdong Provincial People's Hospital (Guangdong Academy of Medical Sciences) Southern Medical University Guangzhou China; ^6^ Engineering Research Center of Bone and Joint Precision Medicine, Department of Orthopedics Peking University Third Hospital Beijing China; ^7^ Department of Bone and Joint Surgery, Department of Orthopedics, Renji Hospital Shanghai Jiaotong University School of Medicine Shanghai China; ^8^ The Third Affiliated Hospital of Southern Medical University Southern Medical University Guangzhou Guangdong China

**Keywords:** biodegradable Zn alloys, bone healing, micro‐galvanic, reconfigure immune, zinc ion window

## Abstract

Ion release‐driven inflammation and bone loss underlie metallic implant failure. Here, micro‐galvanic coupling design of alloys electrochemically programs essential element releasing dose to modulate immune responses and promote regenerative healing. Using model alloys, finely dispersed Mg_2_Zn_11_ particles in Zn‐0.8Mg act as sacrificial anodes, suppressing Zn matrix dissolution. In contrast, coarse FeZn_13_ phases in Zn‐0.8Fe establish a “large cathode‐small anode” galvanic regime, elevating anodic current densities by two orders of magnitude. Consequently, Zn‐0.8Fe implants in bone marrow release excessive Zn^2+^, drive massive neutrophil recruitment and proinflammatory polarization (Day 3), and induce M1‐dominant macrophage responses (Day 14), culminating in poor bone formation at Day 90. Conversely, Zn‐0.8Mg releases a moderate Zn^2+^ flux, promotes pro‐regenerative neutrophil polarization, and guides an M2‐dominant macrophage milieu that fosters osteogenesis rich in type I collagen. Findings here provide the composition design rationale for the development and translational application of a biodegradable Zn alloy cranio‐maxillofacial internal fixation system.

## Graphical abstract

This study utilized alloy micro‐galvanic coupling design to regulate the release of essential elements, thereby programming immune responses and promoting regeneration. The sacrificial anodic process of Zn‐0.8Mg reduced Zn^2+^ release compared to the “large cathode‐small anode” coupling of Zn‐0.8Fe. It guided regenerative neutrophil polarization and the M2 macrophage environment, promoting osteogenic formation rich in type I collagen.

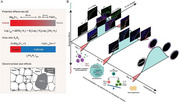

## 1 Introduction

Metallic implants are widely used in orthopedic clinics. Even highly corrosion‐resistant and chemically stable metals such as cobalt (Co) and titanium (Ti) alloys are susceptible to electrochemical corrosion under cyclic stress and micromotion, resulting in local ion release [1, 2]. The released metal ions and wear particles can activate immune cells, including macrophages, to secrete pro‐inflammatory cytokines such as TNF‐α and IL‐1β. These cytokines stimulate osteoclast differentiation and bone resorption, leading to peri‐implant bone loss and aseptic loosening [3, 4]. Over time, this process exacerbates pain and often necessitates revision surgery. In addition, certain patients exhibit delayed‐type hypersensitivity (T‐cell‐mediated) or other immune responses to specific metals such as Co, nickel (Ni), and chromium (Cr) [5, 6]. In these cases, metal ions act as sensitizing antigens, triggering localized or systemic immune reactions. Therefore, traditional bioinert metallic implants are designed or surface‐modified to suppress ion release, thereby mitigating inflammation‐induced implant failure [7, 8]. However, essential metal ions play a crucial role in bone immunoregulation. A variety of bioactive metallic elements, including Zinc (Zn), magnesium (Mg), strontium (Sr), copper (Cu), manganese (Mn), and iron (Fe) have been demonstrated to modulate the bone immune microenvironment and osteogenic processes through distinct signaling pathways [9, 10, 11, 12, 13, 14]. Nevertheless, their biological effects are highly dose‐dependent, as excessive ion release can induce oxidative stress and inflammatory bone resorption.

Biodegradable Zn alloys are highly promising candidates for bone repair applications in their ability to degrade and be metabolized in vivo [15, 16]. This eliminates the need for secondary surgical removal, thereby alleviating the associated physical and financial burdens on patients. Moreover, through appropriate alloying, Zn alloys can be engineered to achieve mechanical properties, including yield strength, ultimate tensile strength, and elongation, that meet the requirements for medical implants, matching the performance of traditional biomaterials like Ti alloys and 316L stainless steel [17]. While pure Zn can fully degrade and be absorbed in a femoral condyle environment, the resulting excess Zn ion (Zn^2+^) can trigger a more intense local inflammatory response compared to inert Ti [18]. Interestingly, the release kinetics of Zn^2+^ can be strategically regulated through both alloy composition and structural design. Extensive researches indicate that incorporating elements such as Li, Mg, and Mn can reduce the degradation rate of Zn alloys, thereby attenuating Zn^2+^ release [19, 20, 21]. Alternatively, structural modulation, as seen in Zn alloy scaffolds with high porosity (e.g., 90%) and large specific surface area, can control local Zn^2+^ concentrations by altering diffusion rates. Consequently, such designed alloys and scaffolds have demonstrated excellent new bone formation and effective osseointegration in vivo [22]. Inspired by this, we propose a paradigm shift: rather than simply suppressing ion release to avoid inflammation, as is the case for traditional bioinert metals, the bioactivity of released ions can be harnessed to actively modulate the immune cascade and, in turn, enhance both the efficiency and quality of bone regeneration.

More importantly, Zn^2+^ plays a pivotal role in *osteoimmunomodulation*. Within the optimal local concentrations, Zn^2+^ bridges the immune and skeletal systems by simultaneously modulating immune cells (such as macrophages) and supporting osteogenic cells (osteoblasts and mesenchymal stem cells) [23, 24], ultimately influencing the balance between bone formation and bone resorption. In vitro studies have shown that Zn^2+^ optimally induce macrophage polarization from the M0 to the M2 pro‐healing phenotype within a concentration range of approximately 11.25–45 µM [18], while osteogenic promotion occurs most effectively within 1–50 µM [25]. Lower concentrations show negligible effects, whereas excessively high concentrations (hundreds of µM) lead to cytotoxicity, pro‐inflammatory responses, and inhibition of osteogenesis [26, 27]. Mechanistically, Zn^2+^ interferes with Toll‐like receptor and LPS‐induced NF‐κB activation, thereby reducing the expression of pro‐inflammatory cytokines such as TNF‐α and IL‐6 [28]. Moreover, Zn^2+^ modulates macrophage polarization through JAK‐STAT signaling pathways and by regulating cellular redox balance and ROS levels, promoting the M2 phenotype characterized by IL‐10 and Arg1 expression and the secretion of pro‐osteogenic and pro‐angiogenic factors [22]. Therefore, an appropriate therapeutic window of Zn^2+^ is essential for achieving balanced osteoimmune modulation.

In biodegradable Zn alloys, micro‐galvanic couples formed between the precipitated second phase and the matrix fundamentally alter the electrochemical dissolution kinetics, as the degradation is governed by the potential difference and the area ratio between the anodic and cathodic phases. To harness this electrochemical principle, Mg and Fe were strategically chosen as alloying elements, due to the potentials of the secondary phases they formed are situated on opposite sides of the Zn matrix [29, 30, 31]. Furthermore, it has been preliminarily established that they elicit significantly different osteogenic effects in vivo. Specifically, Zn‐Mg alloys demonstrate enhanced osteogenic capability compared to pure Zn, while Zn‐Fe alloys conversely show a diminished effect [19, 32]. To ensure their concentrations exceed the solid solubility limits within the Zn matrix, the precipitation of different second phases is effectively promoted (Figure S1A, B). In addition, since the surface area ratio between the Zn matrix and the second phase is a key determinant of degradation, the Zn matrix is homogenized using an equal channel angle compression (ECAP) process. This structural homogenization is crucial for directly and rigorously comparing the microelectrode coupling effects from different second phases.

Based on these well‐defined microstructural models, this study aimed to elucidate how micro‐galvanic corrosion in Zn alloys governs local Zn^2+^ release kinetics to regulate immune‐driven bone regeneration. To this end, specific fluorescent probes were employed to directly detect and spatially track free Zn^2+^ within the implant microenvironment, as opposed to total Zn content. Alloy rods were implanted in the femoral medullary cavity to assess their osteogenic and immunomodulatory effects. This site provides a physiological low osteogenic microenvironment, which effectively distinguishes the osteogenic capacity of bioinert Ti from that of bioactive Zn alloys. Furthermore, a realistic traumatic defect model (rabbit femoral condyle fracture) stabilized with Zn alloy screws was used to evaluate the enhancement of fracture healing. Furthermore, this work also precisely linked the micro‐galvanic couples governed local concentration of Zn^2+^ to the response of key immune cells (neutrophils and macrophages), thereby providing a comprehensive assessment of the impact on bone repair.

## 2 Results

### 2.1 Micro‐Galvanic Couples in Zn‐0.8Mg and Zn‐0.8Fe Model Alloys

**FIGURE 1:**
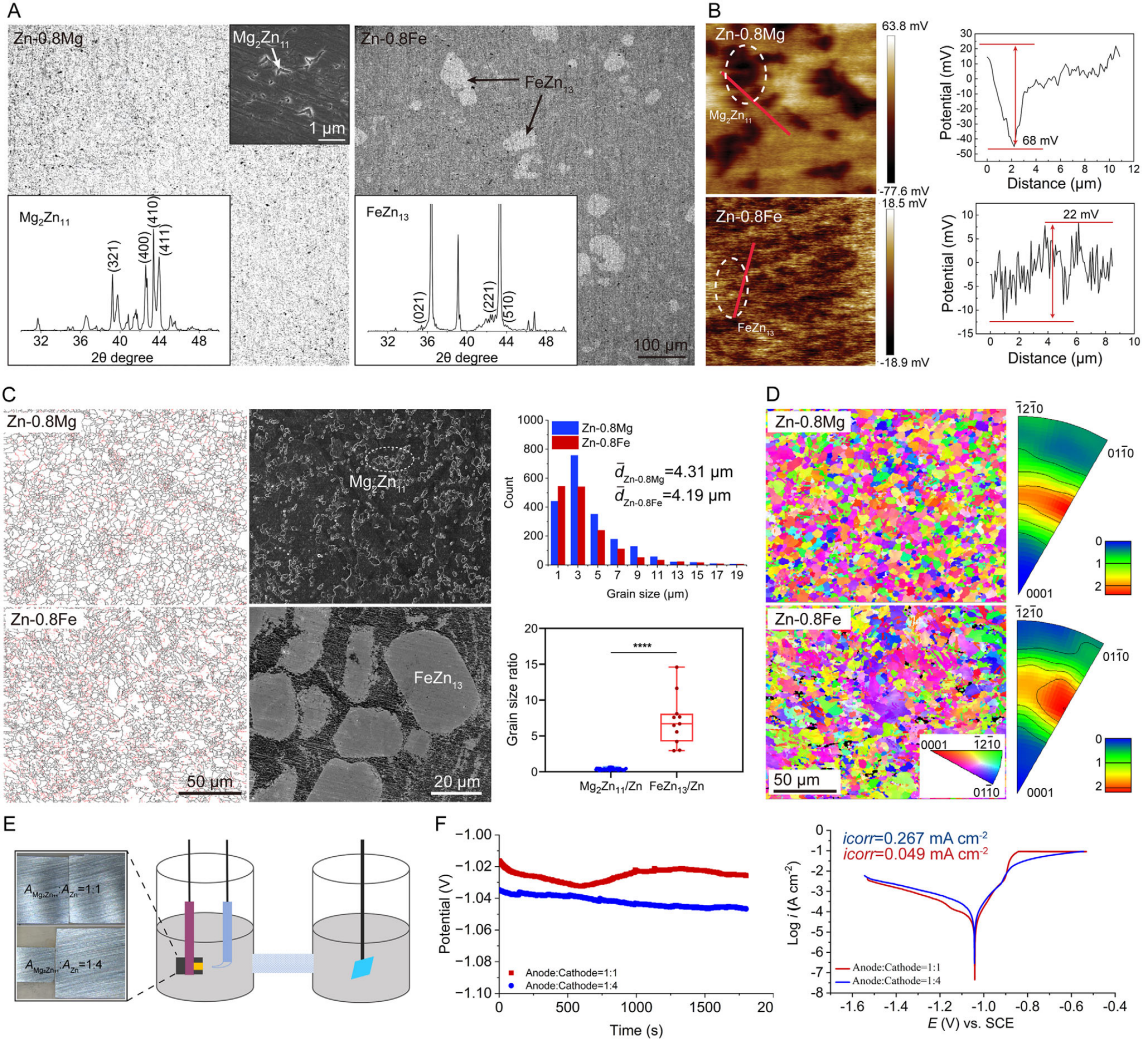
Characterization of micro‐galvanic couples in Zn‐Mg and Zn‐Fe alloys. (A) Backscattered electron images (BSE) and X‐ray diffractions (XRD) of second phase morphology and distribution. (B) Surface potential characterization of second phases and matrix via atomic force microscope (AFM). The left panel displays potential distribution maps, while the right panel quantifies potential differences among distinct phases. (C) Grain boundary quantification and grain size ratio between second phases and Zn (*n* = 25 for Zn‐0.8Mg, *n* = 11 for Zn‐0.8Fe). P‐values were calculated using using an unpaired two‐tailed Student's t‐test, *p < 0.05, **p < 0.01, ***p < 0.001, ****p < 0.0001. Grain boundaries with misorientation angles greater than 15° and less than 15° are delineated in black and red, respectively. (D) Electron Backscatter Diffraction (EBSD) orientation maps and inverse pole figure (IPF). (E) Electrochemical test on pure Mg_2_Zn_11_ phase and Zn phase with different area (*A*) ratio in simulated body fluids (SBF) and (F) Open circuit potential (OCP) and potential dynamic polarization (PDP) curves.

To enable a direct and rigorous comparison of the micro‐galvanic couples from different second phases, the Zn matrix was homogenized via equal‐channel angular pressing (ECAP) (Figure S1C). The Mg_2_Zn_11_ phase in Zn‐0.8Mg alloy was greatly refined into several micrometers and uniformly distributed along Zn grain boundaries (Figure 1A). In contrast, FeZn_13_ phase in tens of micrometers embedded uniformly in the Zn matrix. The surface potential measured by Atomic force microscopy (AFM) revealing that the potential difference between Mg_2_Zn_11_ and Zn is three times greater than that of FeZn_13_ and Zn (Figure 1B). For pure Zn, potential difference mainly occurred between grain boundaries and grain interiors (Figure S2A).

Apart from potential differences, the area ratio between cathode and anode is another critical kinetic parameter in electrochemical reactions. The average grain sizes of matrix were 4.31 and 4.19 µm in Zn‐0.8Mg and Zn‐0.8Fe alloys, respectively, based on grain boundary quantification (Figure 1C). As a result, the calculated area ratio of cathode/anode in Zn‐0.8Mg and Zn‐0.8Fe alloys were 3:1 and 7:1, respectively. In addition, the crystallographic texture orientation and intensity in both alloys are similar (Figure 1D), thereby minimizing microstructural variables unrelated to the alloying effect. To further elucidate the impact of cathode/anode ratio in degradation kinetics, a pure Mg_2_Zn_11_‐phase electrode was fabricated and coupled with pure Zn to form a galvanic macrocell for potential dynamic polarization (PDP) testing (Figure 1E, Figure S3). By varying the cathode‐to‐anode area ratio from 1 to 4, the corrosion current density increased proportionally by a factor of 5.6 (Figure 1F), indicating the linear positive correlation between cathode/anode area ratio and degradation kinetics.

### 2.2 Degradation Kinetics Tailored by Micro‐Galvanic Couples

**FIGURE 2:**
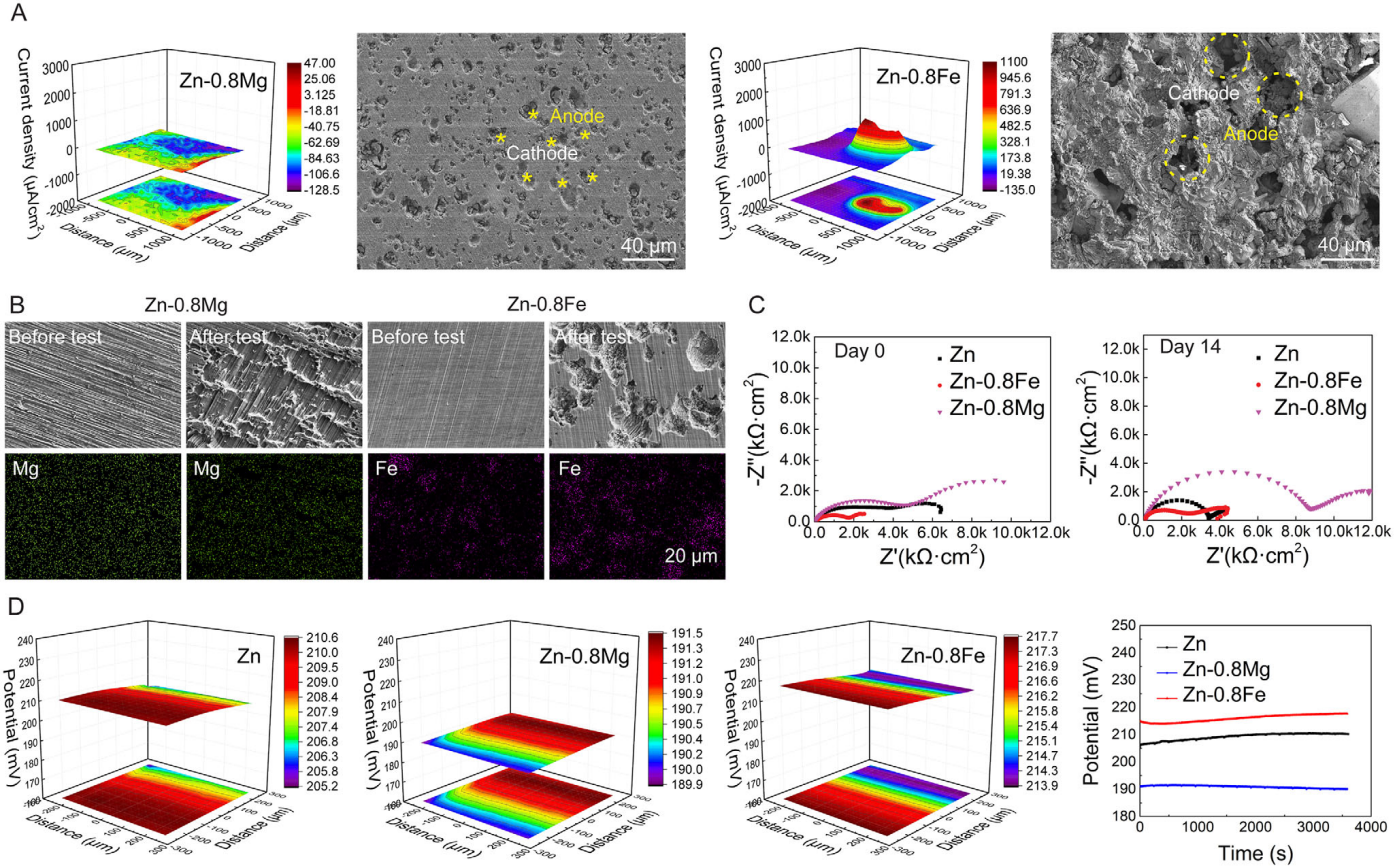
Degradation kinetics of Zn‐0.8Mg and Zn‐0.8Fe alloys. (A) SVET mapping of surface current density distribution after immersion in SBF for 24 h and corrosion morphology after immersed in saline with pH = 4 for 7 days. (B) SEM and EDS mapping of corrosion surface after PDP test. (C) Nyquist and Bode plots of EIS for unimmersed samples and after 14 days of immersion in SBF. (D) SIET diagrams and quantitative curves to reflect the concentration change of released ions during the degradation process of Zn alloy.

The samples were immersed in simulated body fluid (SBF), and scanning vibrating electrode technique (SVET) was employed to map the current density distribution generated by electrochemical reactions on the sample surface (Figure 2A). Compared with pure Zn (Figure S2B), both Zn‐0.8Mg and Zn‐0.8Fe alloys exhibited pronounced local galvanic coupling. For the Zn‐0.8Mg alloy, both cathodic and anodic current densities increased by approximately 4‐fold relative to pure Zn, whereas the anodic current density of the Zn‐0.8Fe alloy increased by nearly 150‐fold. In degradation morphology, the Zn‐0.8Mg alloy displayed uniformly distributed corrosion pits, attributed to the preferential dissolution of the Mg_2_Zn_11_ phase. In contrast, the FeZn_13_ phase in the Zn‐0.8Fe alloy accelerated the electrochemical dissolution of the Zn matrix, resulting in the most severe degradation. Tafel extrapolation analysis revealed that the corrosion current density of the Zn‐0.8Mg alloy was reduced by 33% compared to pure Zn, while that of the Zn‐0.8Fe alloy increased by 65% (Table S1). Elemental mapping of the degraded surfaces indicated localized Mg depletion on the Zn‐0.8Mg alloy after potentiodynamic polarization (PDP) testing, whereas the surface coverage of Fe increased on the Zn‐0.8Fe alloy (Figure 2B).

Furthermore, electrochemical impedance spectroscopy (EIS) was used to monitor the time‐dependent evolution of the corrosion layer on the alloy surfaces in SBF (Figure 2C). From the results obtained immediately immersion and after 14 days, a significant increase in surface impedance was observed for the Zn‐0.8Mg alloy, whereas pure Zn and Zn‐0.8Fe alloy exhibited negligible changes. Ion‐selective electrode technique (SIET) measurements revealed that the increase in surface ion concentration due to degradation followed the order Zn‐0.8Fe > Zn > Zn‐0.8Mg (Figure 2D).

### 2.3 Early Inflammatory Responses Induced by Zn Alloy Implants at Day 3

**FIGURE 3:**
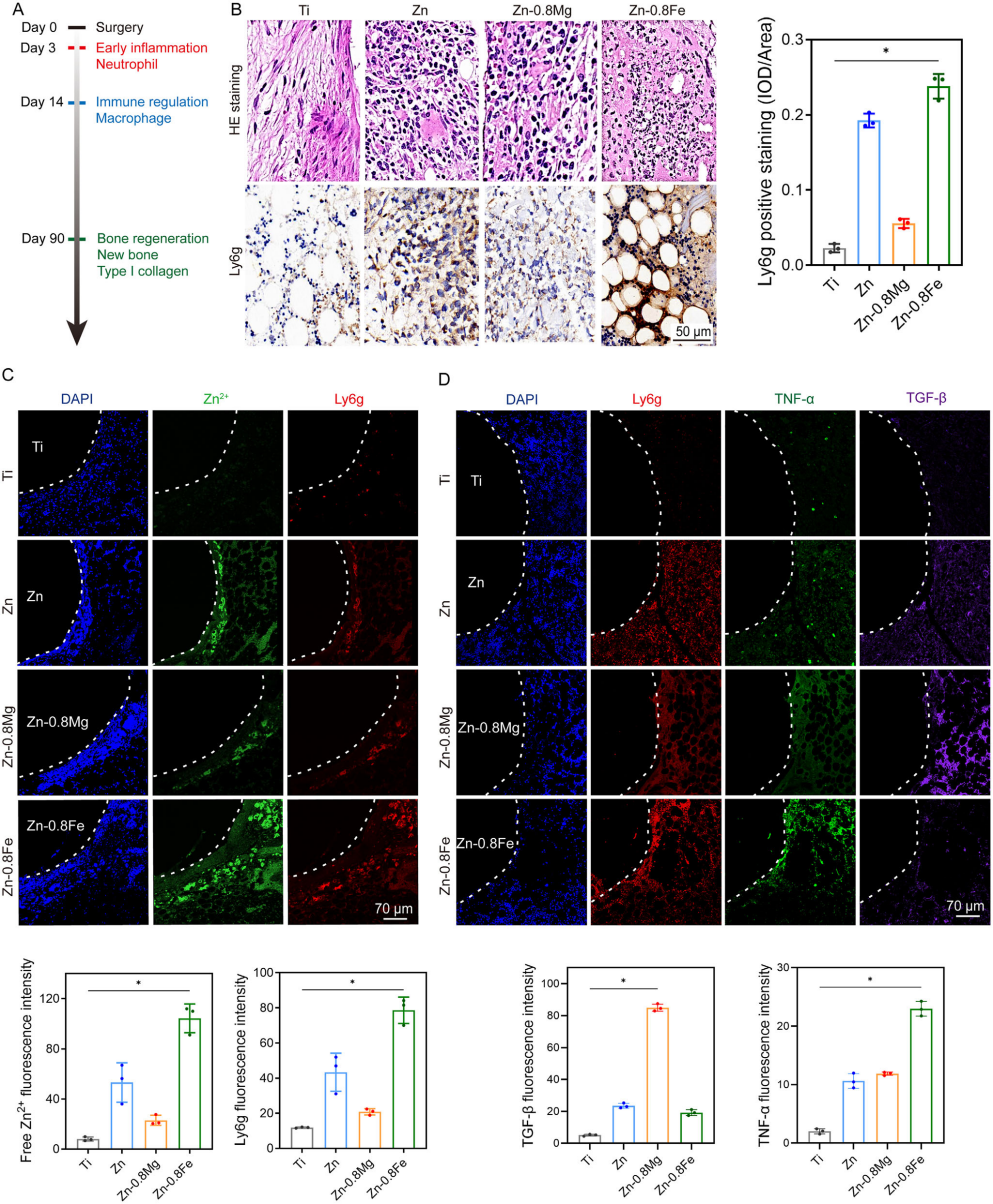
Early inflammation induced by Zn alloy implants at Day 3. (A) Selected time points and events in rabbit femoral shaft critical bone defect model. (B) Neutrophil immunohistochemistry staining and corresponding semi‐quantitative analysis (*n* = 3, independent samples). (C) Tissue free Zn^2+^ and neutrophil marker staining to in early inflammation and corresponding semi‐quantitative analysis (*n* = 3, independent samples). DAPI (blue), Zn^2+^ (green), Ly6g (red). The lower panels show semi‐quantitative analysis of free Zn^2+^ and Ly6g (*n* = 3, independent samples). (D) Neutrophil phenotype determination. Ly6g (red), N1 is pro‐inflammatory type with high expression of TNF‐α (green), while N2 is anti‐inflammatory type with high expression of TGF‐β (purple). The lower panels show semi‐quantitative analysis of TGF‐β and TNF‐α positive regions (*n* = 3, independent samples). Data are presented as mean ± SD. P‐values were calculated using the Kruskal‐Wallis test with Dunn's post hoc test, *p < 0.05, **p < 0.01, ***p < 0.001, ****p < 0.0001.

A rabbit femoral bone defect model was established to investigate the immune responses of the bone microenvironment to Zn alloy implants during in vivo degradation, as well as the subsequent bone regeneration process. The study focused primarily on the early neutrophil‐dominated inflammatory response and the macrophage‐mediated immunoregulatory phase (Figure 3A). Hematoxylin and eosin (H&E) staining, together with immunohistochemical staining for the neutrophil marker Ly6g, revealed a higher recruitment of neutrophils around the pure Zn and Zn alloy implants compared with the medical‐grade Ti group, following the order: Zn‐0.8Fe > Zn > Zn‐0.8Mg > Ti (Figure 3B). Further, in situ co‐staining using a Zn^2+^ fluorescent probe and Ly6g immunofluorescence demonstrated a positive correlation between local Zn^2+^ concentration released from the degrading implants and the extent of neutrophil recruitment in the surrounding tissue (Figure 3C). In in vitro experiments, co‐culturing neutrophils with 100% extract solutions from the materials revealed that Zn alloy extracts significantly promoted neutrophil proliferation (Figure S4).

Further immunofluorescence staining was conducted to investigate the functional polarization of neutrophils (Figure 3D). It was found that neutrophils surrounding the Zn‐0.8Mg and pure Zn implant sites predominantly exhibited a pro‐reparative phenotype (TGF‐β^+^), whereas those around the Zn‐0.8Fe implants mainly displayed a pro‐inflammatory phenotype (TNF‐α^+^). Compared with the pure Ti group, both pure Zn and Zn alloy implants elicited a markedly stronger early immune response. To examine whether neutrophil phenotypes were influenced by Zn^2+^ concentration, neutrophils were co‐cultured with serially diluted extracts of pure Zn (Figure S5). The results demonstrated a dose‐dependent effect of Zn^2+^ on neutrophil functional polarization. Specifically, low concentrations of Zn^2+^ significantly upregulated the expression of pro‐reparative genes (Arg1 and TGF‐β), whereas high concentrations of Zn^2+^ enhanced the expression of pro‐inflammatory genes (iNOS and IL‐1β).

### 2.4 Immune Regulation Induced by Zn Alloy Implants at Day 14

**FIGURE 4:**
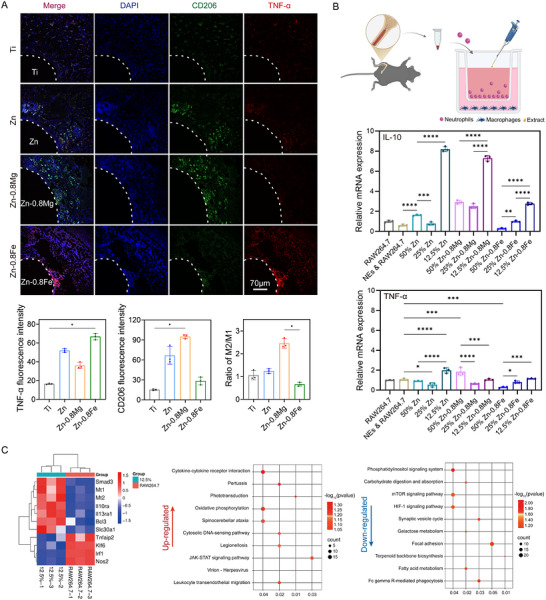
Immune regulation induced by Zn alloy implants at Day 14. (A) Representative immunofluorescence images showing M1 macrophages (TNF‐α) and M2 macrophages (CD206) in tissue sections. Semi‐quantitative analysis of TNF‐α‐ and CD206‐positive cells is shown in the lower panels (*n* = 3, independent samples). P‐values were calculated using the Kruskal‐Wallis test with Dunn's post hoc test. (B) The modulatory effect of primary neutrophils on macrophages under gradient diluted alloy extracts was investigated using an indirect co‐culture system. The lower panels show RT‐PCR results for TNF‐α and IL‐10 expression associated with macrophage polarization (*n* = 3, independent samples). RAW264.7 served as the control group, NEs & RAW264.7 represented the co‐culture without the addition of the extract, and the remaining groups consisted of co‐cultures supplemented with the extract at various concentrations. P‐values were calculated using one‐way ANOVA with Tukey's post hoc test. (C) Heatmap depicting the expression patterns of 11 representative differentially expressed genes between the 12.5% Zn‐0.8Mg extract‐treated group and the control group (*n* = 3, independent samples). Significantly upregulated and downregulated signaling pathways identified by KEGG pathway analysis. P‐values were calculated using two‐sided t‐tests without adjustment. Data are presented as mean ± SD. *p < 0.05, **p < 0.01, ***p < 0.001, ****p < 0.0001.

Next, we examined the influence of Zn alloy implants on the macrophage‐dominated immune microenvironment at 14 days post‐implantation. Immunofluorescence staining of typical macrophage phenotype markers revealed that macrophages surrounding the pure Zn and Zn‐0.8Mg implants predominantly exhibited an M2 pro‐reparative phenotype, with the Zn‐0.8Mg group showing the strongest CD206 fluorescence intensity (Figure 4A). In contrast, the Zn‐0.8Fe group was dominated by M1 pro‐inflammatory macrophages, characterized by the highest TNF‐α fluorescence intensity. The pure Ti group showed the lowest fluorescence intensities for both markers. Among all groups, the Zn‐0.8Mg alloy exhibited the highest M2/M1 ratio (≈2.5), while the Zn‐0.8Fe group remained at approximately 0.5, indicating that the Zn‐0.8Mg alloy had transitioned to the tissue regeneration phase, whereas the Zn‐0.8Fe alloy remained in a pro‐inflammatory state.

To systematically investigate the impact of Zn alloy degradation on the modulation of the immune microenvironment, an in vitro indirect co‐culture model was established by incubating primary neutrophils (isolated from mouse bone marrow) with RAW264.7 macrophages (Figure 4B). Gradient concentrations of alloy extracts (50%, 25%, and 12.5%) were selected based on preliminary cytotoxicity assays to evaluate their immunomodulatory effects (Figure S6). The results showed that mRNA expression of M1‐type pro‐inflammatory markers (TNF‐α) was not concentration‐dependent, whereas M2‐type anti‐inflammatory markers (IL‐10) decreased in a dose‐dependent manner. Low concentrations of Zn^2+^ significantly promoted polarization toward the M2 phenotype, suggesting that the Zn^2+^ dose released during early degradation plays a critical role in optimizing the inflammatory microenvironment. Moreover, MC3T3‐E1 cells were cultured for 14 days in conditioned media which from the indirect co‐culture of different Zn alloy groups. The results demonstrated that conditioned medium containing low concentrations of Zn^2+^ promoted osteogenic mineralization (Figure S7). This direct phenotypic evidence strongly substantiated that, when the release of Zn^2+^ was controlled, a coordinated anti‐inflammatory immune microenvironment effectively promoted osteogenesis.

To further elucidate how Zn alloy extracts influence neutrophil‐mediated macrophage polarization, RAW264.7 macrophages were cultured with an eightfold diluted extract of the Zn‐0.8Mg alloy and subjected to transcriptomic analysis. Bioinformatic analysis identified 202 differentially expressed genes (DEGs) between the two groups, including 58 upregulated and 144 downregulated genes. The volcano plot highlighted pronounced transcriptomic heterogeneity induced by the extract (Figure S8A). Hierarchical clustering revealed a distinct expression module comprising the top 11 hub genes, characterized by upregulation of M2‐associated markers (e.g., IL‐10, IL‐13) and suppression of M1‐related genes (e.g., IL‐1β, Nos2) (Figure 4C).

Gene Ontology (GO) enrichment analysis indicated that the DEGs were primarily involved in biological processes such as cell proliferation, cytoskeletal remodeling, and immune regulation, suggesting that the Zn‐0.8Mg alloy extract promotes M2 polarization by modulating cellular morphology and immunometabolic signaling (Figure S8B). KEGG pathway enrichment analysis further revealed that upregulation of key components within the JAK‐STAT signaling pathway was positively correlated with M2 macrophage polarization, whereas genes associated with the HIF‐1 signaling pathway were significantly downregulated.

### 2.5 Bone Regeneration Induced by Zn Alloy Implants at Day 90

**FIGURE 5:**
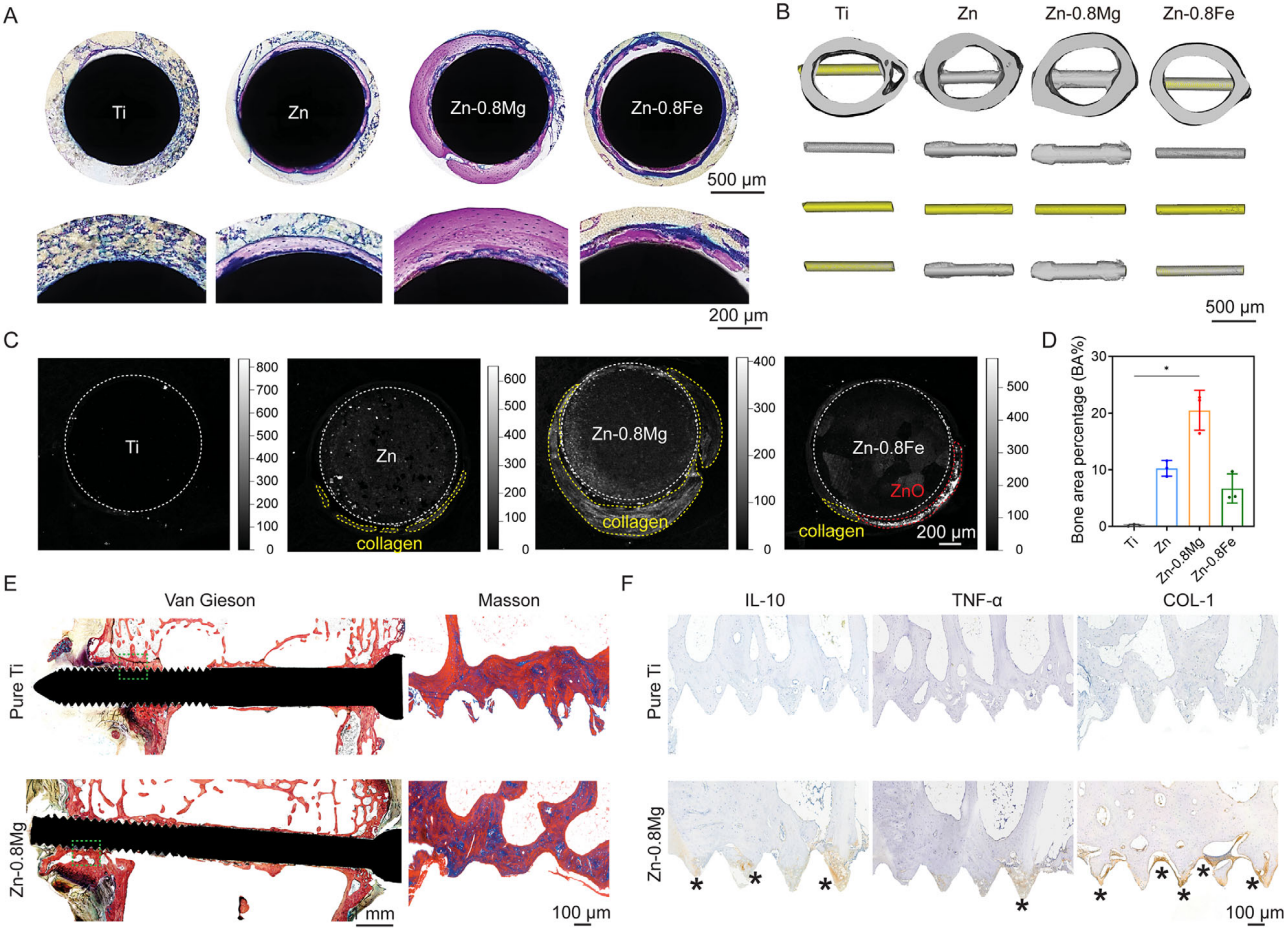
Bone regeneration induced by Zn alloy implants at Day 90. (A) Methylene blue‐fuchsin staining of undecalcified hard tissue sections of pure Ti, pure Zn, Zn‐0.8Mg, and Zn‐0.8Fe alloy implants with bone regeneration. (B) Micro‐CT three‐dimensional reconstruction showing metallic implants (yellow), newly formed bone tissue (silver‐gray) and combination. (C) SHG imaging of the implantation sites at 3 months post‐surgery. Yellow dotted regions indicate type I collagen fiber, while red dotted areas highlight ZnO. (D) Bone area percentage (BA%) calculated based on backscattered electron (BSE) images of undecalcified hard tissue sections (*n* = 3, independent samples), a region of interest (ROI) with a diameter of 1.4 mm. P‐values were calculated from the non‐parametric Kruskal‐Wallis test, followed by Dunn's test for multiple comparisons. *p < 0.05, **p < 0.01, ***p < 0.001, ****p < 0.0001. (E) Representative image of Van Gieson, Masson in rabbit femoral tissue sections after 3 months implantation of pure Ti and Zn‐0.8Mg alloy screws. (F) Immunohistochemical staining of IL‐10 (anti‐inflammatory cytokines), TNF‐α (pro‐inflammatory cytokines) and COL‐1 (osteogenic‐related makers). Positive areas are marked by asterisks.

To comprehensively assess the osteogenic capability of Zn alloys, a multimodal evaluation strategy was employed, including methylene blue‐acid fuchsin staining, second‐harmonic generation (SHG) imaging, and micro‐computed tomography (micro‐CT) to characterize hard tissue regeneration. As shown in Figure 5A and B, because the medullary cavity is predominantly composed of non‐osteogenic marrow tissue, the chemically stable and bioinert pure Ti implants predictably remained largely unintegrated and exposed. In contrast, all Zn alloys implants actively induced new bone formation and were at least partially encapsulated by newly formed bone. Quantitative analysis (Figure 5D) revealed that Zn‐0.8Mg exhibited the most robust osteogenic response compared to pure Ti, with the entire implantation site occupied by newly formed bone in close apposition to the implant surface. Pure Zn also promoted evident bone regeneration and demonstrated favorable bone‐implant contact, albeit to a lesser extent than Zn‐0.8Mg. In contrast, Zn‐0.8Fe showed comparatively inferior osteogenic performance, characterized by reduced new bone volume and suboptimal osseointegration. In this group, direct bone‐implant contact was limited, and large areas displayed clear separation between the implant and adjacent bone tissue.

SHG imaging, a non‐destructive technique for visualizing collagen architecture, offered additional insights into peri‐implant tissue responses (Figure 5C). Two distinct SHG signals were observed: one corresponding to type I collagen fibers within regenerating bone tissue, and the other attributable to ZnO‐related crystalline structures associated with the degradation of Zn implants. Notably, no SHG signal was detected in the vicinity of pure Ti implant, suggesting a lack of collagen remodeling or ZnO formation. In contrast, all Zn alloy groups exhibited clear evidence of type I collagen deposition. Among them, Zn‐0.8Mg demonstrated nearly complete circumferential collagen coverage around the implant, indicative of active tissue remodeling. In the Zn‐0.8Fe group, a strong ZnO‐related signal was concentrated at the implant‐tissue interface, reflecting substantial accumulation of degradation products at three months post‐implantation. Pure Ti implants remained largely unintegrated and exposed within the medullary cavity. By contrast, all Zn alloys implants were at least partially encapsulated by newly formed bone (rendered in gray). The Zn‐0.8Mg group exhibited the most robust bone regeneration, with new bone bridging the implant and adjacent cortical bone, forming a continuous interface indicative of superior osseointegration. As shown in Figure S9, immunohistochemical analysis showed elevated expression of the osteogenic markers alkaline phosphatase (ALP) and osteocalcin (OCN) in all Zn alloy groups relative to the Ti group. The Zn‐0.8Mg alloy group showed the highest upregulation of both ALP and OCN, followed by pure Zn and Zn‐0.8Fe. Collectively, these findings underscore Zn‐0.8Mg alloy as a particularly promising Zn alloys, capable of enhancing osteogenic activity with high quality new bone in vivo.

### 2.6 Rabbit Femoral Tissue Sections After 3 Months Implantation of Pure Ti and Zn‐0.8Mg Alloy Screws

Zn‐0.8Mg alloy exhibits an excellent osteogenic promotion capability. To verify the potential clinical application, a rabbit femoral condyle fracture model was established to evaluate the overall efficacy of the Zn‐0.8Mg alloy internal fixation screw in promoting bone fracture healing, with the widely used pure Ti screw serving as the control. Radiographic analysis confirmed that the fracture lines in both groups were stabilized and completely healed at 3 months post‐surgery (Figure S10). Van Gieson and Masson staining were employed to visualize new bone formation around the screws (Figure 5E). On both sides of the cortical bone, the newly formed bone exhibited close integration with the screw bodies and threads. In the central region of the femoral condyle, more newly formed bone was observed surrounding the Zn‐0.8Mg alloy screws compared with the Ti group. Higher‐magnification Masson staining around the threads revealed a greater amount of mature collagen and more advanced bone matrix mineralization (indicated by blue staining) in the Zn‐0.8Mg group.

To further assess the inflammatory response and osteogenic status of peri‐implant tissues during the mid‐to‐late stages of bone formation, immunohistochemical staining was performed (Figure 5F). In the pure Ti group, virtually no positive expression of the tested markers was detected, indicating the absence of an inflammatory response and no interference with osteogenesis. In contrast, in the Zn‐0.8Mg group, positive expression of the markers (highlighted by asterisks) was observed adjacent to the screw surfaces and within the thread regions, while tissues several millimeters away from the implant surface remained unaffected. Notably, strong IL‐10 expression accompanied by weak TNF‐α expression around the Zn‐0.8Mg screws suggested that the surrounding tissues were in a pro‐regenerative immune microenvironment. Concurrent strong positive expression of COL‐1, OPG and OCN (Figure S11) further demonstrated that this immune milieu suppressed osteoclastogenesis and bone resorption, while simultaneously promoting osteoblast‐mediated matrix protein secretion and bone mineralization.

### 2.7 Hierarchical Multiscale Analysis of the Implant‐Bone Interface

**FIGURE 6:**
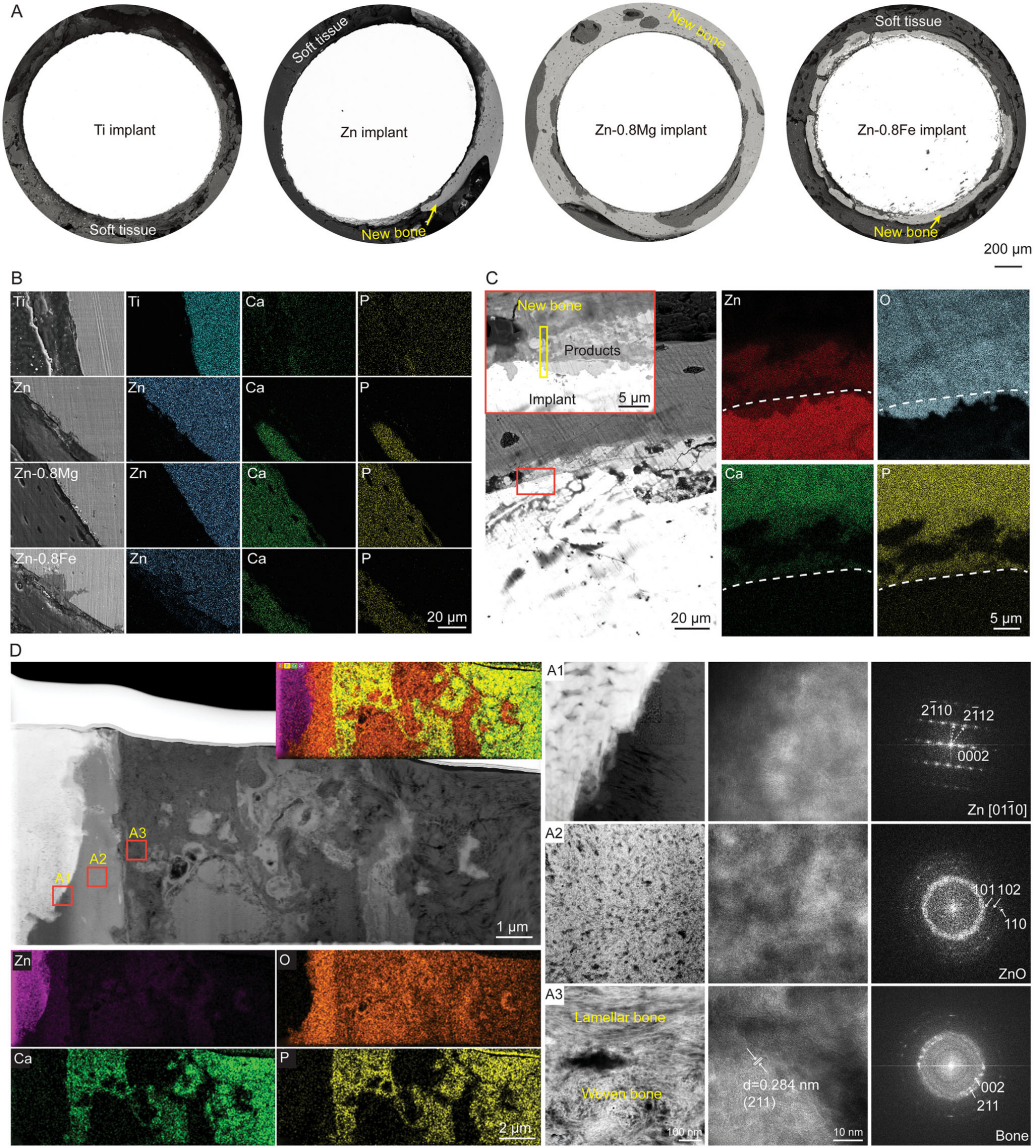
Hierarchical multiscale analysis of the implant‐bone interface. (A) Cross‐sectional BSE images of implants 3 months after surgery. (B) SEM magnification of a representative bone‐implant interface and corresponding elemental mapping. (C) Magnification of a backscatter electron (BSE) image of a representative bone‐implant interface and corresponding elemental mapping. (D) Focused ion beam (FIB) sample preparation and transmission electron microscopy (TEM) characterization with high‐angle annular dark‐field (HAADF) image and corresponding elemental mapping, and high‐resolution TEM (HRTEM) images and selected area electron diffraction (SAED) of hierarchical structure of representative bone‐implant interface.

To further elucidate the potential relationship between Zn alloy degradation and bone regeneration, the elemental and structural characteristics at the bone‐implant interface were analyzed using scanning electron microscopy (SEM) and transmission electron microscopy (TEM). Under BSE imaging, three distinct contrast regions were observed: the brightest region corresponded to the metallic implant, the darkest region represented the surrounding soft tissue, and the intermediate region indicated the newly formed bone. A continuous and well‐organized layer of new bone was clearly visible around the Zn‐0.8Mg alloy implant, whereas the Zn‐0.8Fe group exhibited significantly less and morphologically irregular new bone formation. In contrast, no new bone formation was observed around the Ti implant (Figure 6A).

Energy‐dispersive X‐ray spectroscopy (EDS) mapping of the bone‐implant interface confirmed consistent results (Figure 6B). Examination of a representative continuous interfacial region, including the metallic substrate, degradation layer, and new bone revealed that within the original contour of the Zn alloy (indicated by a white dashed line), the inner corrosion products were mainly composed of Zn and O. The outer degradation layer contained Zn, Ca, P, and O, which gradually transitioned into the newly formed bone tissue dominated by Ca, P, and O (Figure 6C).

A thin lamella (≈ 50 nm thick) was further prepared from the interfacial area (yellow rectangle in Figure 6C) using focused ion beam (FIB) technology and analyzed by TEM to investigate its compositional and structural features (Figure 6D). Based on high‐angle annular dark‐field (HAADF) imaging and elemental mapping, three distinct regions were identified across the lamella from left to right: the metallic matrix, the degradation product layer, and the product‐tissue transition zone.

High‐resolution TEM (HRTEM) and selected area electron diffraction (SAED) analyses revealed clear structural transitions across the interface. In the metal‐product interface region (A1), sharp diffraction spots characteristic of crystalline Zn was observed. The degradation product region (A2) consisted primarily of amorphous material interspersed with ≈ 10 nm clusters, exhibiting diffraction rings corresponding to nanocrystalline ZnO. In the tissue region (A3), rich in Ca and P, two distinct bone morphologies were identified: disordered woven bone and well‐organized lamellar bone, the latter displaying visible collagen fibril bundles embedded with nano‐hydroxyapatite (n‐HA) particles.

Notably, a distinct Zn elemental signal was detected throughout the newly formed bone at the interface. Elemental profiling showed a gradual decrease in Zn concentration from the metallic substrate toward the bone tissue, accompanied by a progressive increase in Ca and P content. These findings indicate that Zn^2+^ released during alloy degradation actively participates in new bone formation, influencing both its chemical composition and microstructural organization.

## 3 Discussion

**FIGURE 7:**
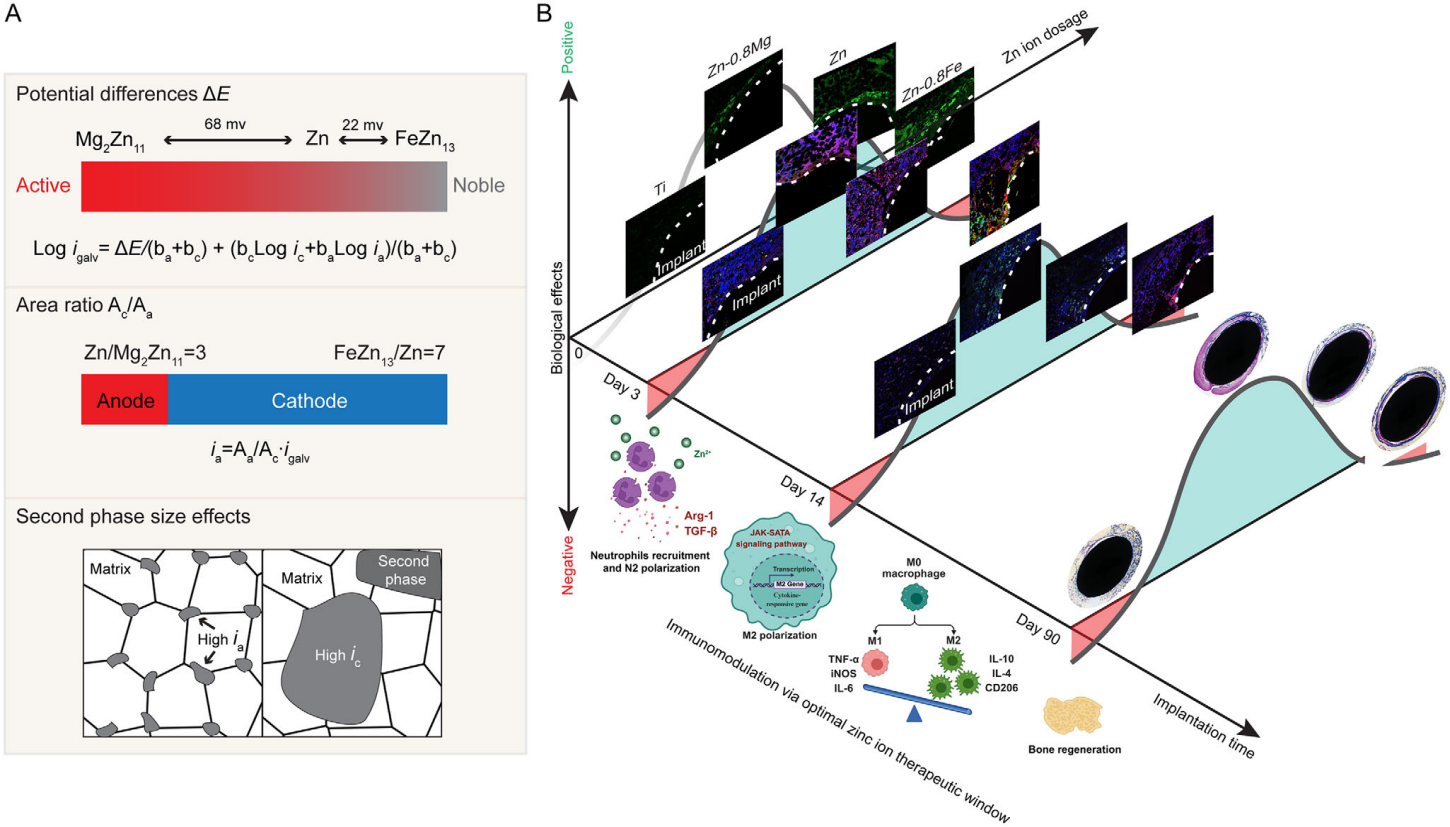
(A) Electrochemical parameters that can be utilized to program the ion releasing kinetics from Zn alloys. (*i*_galv_: galvanic current density, Δ*E*: potential differences between matrix and second phases, *i*_a_: anodic current density, *i*_c_: cathodic current density, *b_a_*: anodic Tafel slope, *b_c_*: cathodic Tafel slope). (B) Zinc^2+^ therapeutic window reconfigures immune cascades for pro‐regenerative bone healing.

In this study, Zn‐Mg and Zn‐Fe alloys were selected as model systems. We found that the addition of alloying elements significantly altered the degradation characteristics of pure Zn. Specifically, incorporation of 0.8 wt.% Mg or Fe led to the formation of secondary phases: Mg_2_Zn_11_ and FeZn_13_, respectively. Within the Zn matrix, generating micro‐galvanic couples due to potential differences between the phases (Figure 7A). Based on the mixed potential theory and the Tafel approximation, the micro‐galvanic current density (*i*_galv_) exhibits a log‐linear relationship with the potential difference (Δ*E*) [33]. Accordingly, engineering the Δ*E* between the secondary phase and the matrix phase directly regulates *i*_galv_ and determines the dissolution kinetics of ions within the phases. In addition, the area ratio between the micro‐galvanic cathode and anode (A_c_/A_a_) serves as a corrective parameter (*Catchment principle* [34]) for the anodic dissolution current as verified in Figure 1E and F. To enable a clear visual comparison of the size difference between the second phase and the Zn matrix, the alloy was subjected to a matrix homogenization treatment via ECAP (Figure 1 and Figure S1). Therefore, the resulting matrix grain sizes for Zn‐0.8Mg and Zn‐0.8Fe were comparable and measured as 4.31 and 4.19 µm, respectively (Figure 1C). The FeZn_13_ phase was approximately seven times larger than the Zn grains (A_c_/A_a_ = 7), forming a large‐cathode/small‐anode configuration that markedly intensified Zn dissolution. Consequently, the anodic current density of Zn‐0.8Fe alloy was nearly 150‐fold higher than that of pure Zn, leading to a significant increase in the local Zn^2+^ concentration. In contrast, the electrochemical dissolution rate of Zn‐0.8Mg was 33% lower than that of pure Zn (Table S1), as the Zn matrix was cathodically protected. To further confirm the critical influence of phase size on degradation rate, we constructed an amplified galvanic corrosion model. It showed that a 4‐fold larger cathode area increased the corrosion current density by 5.6‐fold. This direct correlation indicated a significant difference in Zn^2+^ release: larger cathode area (as in Zn‐0.8Fe) yields higher corrosion current density, thus resulting in a higher release of Zn^2+^. Additionally, the size and spatial distribution of the secondary phase influence micro‐galvanic corrosion by affecting the local electric field distribution and reactant mass transport [35]. When the second phase is fine and dispersed within the matrix, the local anodic current density tends to increase, making localized pitting more likely. In contrast, coarse cathodic phases can sustain higher cathodic polarization currents, thereby accelerating the overall galvanic corrosion rate [36].

The immune response to foreign materials largely determines the fate of biomaterials after implantation. Within the first few days of post‐implantation, neutrophils, serving as early “scavengers” are recruited to the implant site to remove debris and phagocytose foreign materials while secreting cytokines such as TGF‐β and TNF‐α [37, 38]. Subsequently, monocytes and macrophages, the central regulators of immune homeostasis are recruited and polarized [39, 40, 41]. Macrophages play a decisive role in the foreign body response, displaying either a pro‐inflammatory (M1‐like) phenotype (e.g., TNF‐α, iNOS) or a pro‐repair/immunomodulatory (M2‐like) phenotype (e.g., TGF‐β, IL‐10), which collectively govern the balance between fibrosis and tissue regeneration [42, 43, 44]. Zn alloys implants induced a markedly stronger early immune response than pure Ti (Figure 7B). The Zn‐0.8Mg alloy released lower local Zn^2+^ concentrations during degradation, leading to mild neutrophil recruitment and proliferation at day 3 post‐implantation. In contrast, the Zn‐0.8Fe alloy released higher concentrations of Zn^2+^, triggering extensive neutrophil infiltration and activation. Moreover, neutrophil functional polarization was directly modulated by Zn^2+^ concentration. Neutrophils surrounding the Zn‐0.8Mg implants predominantly polarized toward an anti‐inflammatory N2 phenotype, secreting cytokines such as TGF‑β, IL‑10, and IL‑6. Whereas conversely, those near the Zn‐0.8Fe implant adopted a pro‑inflammatory N1 phenotype, associated with pro‑inflammatory cytokine production (e.g., TNF‑α, IFN‑γ, IL‑1) [37]. The Ti group showed minimal neutrophil activation or polarization. Consistent results were obtained in vitro, confirming the dose‐dependent effects of Zn^2+^ on neutrophil proliferation and polarization.

At 14 days post‐implantation, this distinction extended to macrophage functional polarization (Figure 7B). Macrophages surrounding the Zn‐0.8Mg implants were dominated by the M2 (anti‐inflammatory) phenotype, whereas those around Zn‐0.8Fe implants primarily exhibited an M1 (pro‐inflammatory) phenotype. Transcriptomic analysis further revealed that low Zn^2+^ concentrations upregulated key components of the JAK‐STAT signaling pathway, correlating positively with M2 macrophage polarization, while HIF‐1 pathway‐related genes were significantly downregulated. By 90 days post‐implantation, no new bone formation was detected around pure Ti implants, whereas both Zn alloys implants promoted varying degrees of new bone regeneration (Figure 7B). The Zn‐0.8Mg implant was completely surrounded by dense, well‐mineralized new bone rich in type I collagen, with only a thin fibrous interface. In contrast, the Zn‐0.8Fe implant exhibited limited and poorly organized new bone formation with low collagen content, encapsulated by thick fibrous tissue. Finally, in a rabbit femoral condyle fracture model (Figure 5E and F), the Zn‐0.8Mg alloy screws demonstrated superior osteogenic performance compared with pure Ti screws, by establishing a pro‐regenerative immune microenvironment that enhanced osteoblast‐mediated matrix protein secretion and bone mineralization. The Zn‐Mg alloy patent has been granted a Chinese invention patent (patent number: *ZL201410415524.7*) and has subsequently been licensed for the development of Zn alloy orthopedic medical devices. Findings here may also provide the composition design rationale for the development and translational application of biodegradable Zn alloy internal fixation systems for cranio‐maxillofacial surgery (Chinese clinical trial registration number: *ChiCTR2100051050*).

Collectively, this work proposes a design theory for biodegradable Zn alloys, leveraging micro‐galvanic coupling to actively program their therapeutic ion release window. This theory shifts the paradigm from merely inhibiting corrosion to harnessing controlled ion release for immunomodulation, thereby providing conceptual insights. For successful clinical translation, future design of Zn alloys must prioritize strict control over degradation kinetics to ensure that localized Zn^2+^ release remains anchored within this therapeutic window throughout the bone healing cascade. This can be achieved through strategic alloying with osteogenic elements (e.g., Mg, Ca, Sr). By altering the matrix potential or fostering a dense protective layer, the corrosion mode is modulated from localized to uniform. This controlled degradation dilutes the absolute Zn^2+^ concentration, resulting in a more favorable biological microenvironment. Furthermore, precise control over the area ratio and distribution between the second phase and the matrix allows the ion release rate to be tailored to the specific demands of different anatomical sites and healing stages. This tunability thereby enables a new strategy for the precise repair of complex bone defects.

Despite these promising findings, several methodological and translational limitations should be noted. First, although in vitro extraction models clearly characterize the dose‐response relationship of immune cells to Zn^2+^, they are inherently incapable of fully replicating the complex in vivo degradation microenvironment. In reality, the local immune cascade is concurrently influenced by the dynamically changing implant surface topography, localized pH shifts, and the accumulation of solid degradation products like nanocrystalline ZnO and calcium phosphates at the bone‐implant interface. Consequently, although this work strongly support a model in which micro‐galvanic programming of the Zn^2+^ therapeutic window drives pro‐regenerative immunity, the direct causal in vivo sequence must still be inferred. To definitively establish the specific contribution of Zn^2+^ versus concurrent degradation products, future studies employing genetic knockouts or specific pathway inhibitors are needed. Furthermore, while the results demonstrate dynamic changes in the expression of TNF‐α, IL‐10, and TGF‐β, it is important to acknowledge the limited specificity of these markers. These cytokines are prominently secreted by other immune cells, particularly polarized macrophages (M1/M2). Therefore, the observed N1/N2 polarization trends should be interpreted with caution, and future studies utilizing cell‐lineage specific depletion or spatial transcriptomics will be necessary to further dissect the distinct contributions of neutrophil subpopulations. The rabbit femoral defect and fracture models offered valuable in vivo insights, they entailed significant interspecies disparities in immune profiles, baseline bone remodeling rates, and biomechanical loading. These differences consequently constrained direct clinical translation. To fully validate the long‐term clinical efficacy and safety of this micro‐galvanic coupling programed Zn alloy system, future studies must employ large, load‐bearing animal models (e.g., sheep or minipigs) over extended implantation periods. Finally, in strict adherence to the principles of animal welfare, the biological sample size was ethically minimized to *n* = 3 per group. Under conditions of limited sample size, the statistical power is inherently low, and the mathematical assumptions underlying traditional statistical testing (such as normality and equal variance) carry a high degree of uncertainty. Consequently, the quantitative comparisons and any statistical significance derived from the in vivo data should be interpreted with caution. Although these results were supported by consistent cross‐validation outcomes derived from multiple complementary assessment modalities to ensure reliable conclusions, including Micro‐CT, SHG imaging, and comprehensive histological and immunofluorescent assessments. Future studies would benefit from larger sample sizes to enhance statistical power and robustness.

## 4 Conclusions

Collectively, our findings reveal that by engineering compositional and microstructural heterogeneity, it is possible to manipulate micro‐galvanic configurations and thus control Zn^2+^ electrochemical dissolution kinetics. In the Zn‐0.8Mg alloy, the Mg_2_Zn_11_ anodes protect the Zn matrix and maintain Zn^2+^ release within an effective immunoregulatory dose window, enabling the sequential polarization of neutrophils and macrophages toward pro‐reparative phenotypes and ultimately promoting high‐quality bone regeneration. Conversely, the Zn‐0.8Fe alloy induces excessive local Zn^2+^ accumulation, amplifying inflammation and impairing osteogenesis. This study establishes an innovative paradigm in which controlled ion release is reversely exploited as a constructive biological cue to modulate immune responses, thereby achieving immune‐mediated, pro‐regenerative bone healing.

## 5 Methods

### 5.1 Materials Preparation

Pure Zn and Zn alloy ingots were synthesized by Hunan rare earth metal material research institute, the design and actual compositions of all materials were listed in Table S2. Pure titanium (Ti) was purchased as medical grade. After casting, material ingots were homogenized at 350°C for 48 h followed by water quenching. The as‐cast metals were extruded into a rod with a diameter of 10 mm at 200°C, and the extrusion ratio was 16:1. The hot‐extruded rods were divided into short rods with a length of 10 cm and subjected to ECAP at 250°C. Pure Zn and Zn alloys were subjected to 5 and 8 cycles, respectively, without cracks in the material. Except for in vivo experiments, all samples were cut into discs with a thickness of 1 mm. The obtained discs polished to 2000 grit with SiC sandpaper, and then ultrasonically cleaned in anhydrous ethanol. The samples for cell experiments were soaked in 75% ethanol and sterilized by ultraviolet irradiation. The samples for animal experiments were sterilized by ethylene oxide.

### 5.2 Microstructure Characterization

The sample surface perpendicular to the extrusion direction (ED) was polished using 1k eV Ar^+^ ion beam for 30 min with ion milling system (Leica EM RES102). Grain size distribution, phase constituents, grain orientation, and crystallographic texture were characterized by EBSD using a scanning electron microscope (SEM, JSM‐7200F) equipped with an EDAX Velocity Super detector. For surface morphology analysis, the samples were sequentially polished using 5000‐grit SiC paper followed by 0.1 µm diamond suspension. After thorough rinsing with deionized water, the samples were etched in a solution containing 200 g/L CrO_3_ and 15 g/L anhydrous Na_2_SO_4_ to reveal the metallographic structure. Microstructural features and elemental composition were examined using a field‐emission scanning electron microscope (FE‐SEM, Hitachi S‐4800, Japan) equipped with energy‐dispersive X‐ray spectroscopy (EDX). The sample for TEM testing was thinned to approximately 50 nanometers with FIB. Bright‐field images, high‐resolution images, and SAED patterns were pictured by an FEI TEM. The phase composition was analyzed by a CuKα X‐ray diffractometer (XRD, Rigaku DMAX 2400, Japan) at 40 kV and 100 mA. Scanning was performed at a rate of 2 ° min^−1^ in the range of 10 ° to 90 ° with a step size of 0.02 °. The surface potential of pure Zn and Zn alloys and the potential difference between the second phase and the Zn substrate were obtained by the KPFM mode of atomic force microscope (AFM, Bruker Dimension ICON, Bruker, German).

### 5.3 Electrochemical Characterization

The corrosion kinetics of the materials were evaluated using a standardized electrochemical characterization protocol in SBF (NaCl 8.035 g·L^−1^, NaHCO_3_ 0.355 g·L^−1^, KCl 0.25 g·L^−1^, K_2_HPO_4_·3H_2_O 0.231 g·L^−1^, MgCl_2_·6H_2_O 0.311 g·L^−1^, HCl (36%–38%) 39 mL·L^−1^, CaCl_2_ 0.292 g L^−1^, Na_2_SO_4_ 0.072 g·L^−1^, Tris 6.118 g·L^−1^, pH = 7.4). The samples were Zn alloys without immersion and with SBF solution immersion for 14 days (with degradation products retained). The test procedure strictly follows the ASTM standard: first, open‐circuit potential time domain monitoring was carried out to establish the steady state of the system, and then the polarization curve was obtained by using the dynamic potential scanning mode (±500 mV vs. OCP, 1 mV·s^−1^) and the characteristic electrochemical parameters were analyzed by Tafel extrapolation method. Experiments were performed five times in parallel to ensure data reliability.

The effect of anode and cathode dimensions on galvanic corrosion was simulated by varying the surface areas of pure Zn and Mg_2_Zn_11_. Specifically, Mg_2_Zn_11_ cubes with side lengths of 1 cm and 0.5 cm were prepared, while keeping the Zn size at 1 cm. Zn and Mg_2_Zn_11_ were bonded together using conductive silver paste and externally connected with copper sheets. After embedding the connected anode and cathode in resin to form electrodes, electrochemical tests were performed using a three‐electrode system.

### 5.4 Degradation Characterizations

Accelerated corrosion tests were performed by immersion in saline at pH = 4.0 for 1 week, during which the solution was changed when pH > 7. After the immersion test, the corrosion products were removed with CrO_3_ solution and the surface corrosion morphology was observed by SEM. Spatially resolved measurements of micro‐area electrochemical behavior were achieved by SVET. Using an SVET system from Applicable Electronics Inc. (USA), the test was also performed in SBF at room temperature for 24 h with a step size of 100 µm. The release of Zn ions was tested by Scanning Ion‐selective electrode Technique (SIET) under this system.

### 5.5 Cell Experiment

#### 5.5.1 Preparation of Standardized Material Extracts

Pure Zn and Zn alloy extracts were prepared following the ISO 10993‐12 standard. Both sides of the samples were UV‐sterilized for 30 min and incubated in the respective cell culture medium (1.25 cm^2^/mL) at 37°C for 24 h. The collected supernatant served as the 100% extract, from which 50%, 25%, and 12.5% dilutions were freshly prepared prior to cell exposure. The concentrations of metal elements in the extracts of pure Zn and Zn alloys were shown in Figure S4B.

#### 5.5.2 Cytotoxicity Evaluation (CCK‐8 Assay) and Intracellular Zn^2^^+^ Imaging

HL‐60 cells (human acute promyelocytic leukemia, *RRID*: *CVCL_0002*) and RAW264.7 cells (mouse macrophages, *RRID*: *CVCL_0493*) were used to assess basic cytotoxicity. Prior to testing, HL‐60 cells were differentiated into neutrophil‐like cells by culturing in RPMI‐1640 supplemented with 1.5% DMSO for 5 days. Both cell lines were seeded in 96‐well plates (5 × 10^4^ cells/mL) in media containing 10% FBS and 1% penicillin/streptomycin. After 24 h of attachment, the medium was replaced with material extracts of varying concentrations, using standard medium as the negative control. Cell viability was measured at 24 or 48 h using a CCK‐8 kit (Dojindo, Japan) at 450 nm. Three independent experiments were performed for each group, with three replicate wells per condition in each experiment. To track intracellular free Zn^2+^, these primary cells were cultured with 100% Zn alloy extracts for 48 h. Cells were then fixed, permeabilized, and stained with FluoZin‐3 AM for 30 min and DAPI for 10 min. Imaging was performed using a Dragonfly 200 confocal microscope.

#### 5.5.3 Primary Neutrophil Isolation

Primary neutrophils were isolated from the bone marrow of 6–8‐week‐old mice using a BeaverBeads magnetic separation kit (70907‐50) after red blood cell lysis. Flow cytometry was used to test three independent samples of the modified FITC‐anti‐Ly6g and PE‐anti‐CD11b, confirming a purity of 94.1% (Figure S12).

#### 5.5.4 Neutrophil Phenotype Analysis

Isolated primary neutrophils were exposed to 100%, 50%, 25%, and 12.5% pure Zn extracts. After 24 h of exposure, total RNA was extracted. The relative expression of N1 (iNOS, IL‐1β) and N2 (Arg1, TGF‐β) markers was quantified by RT‐PCR, each treatment was tested in three replicates across three independent experiments. Primer sequences used in this study were listed in Table S3.

#### 5.5.5 Neutrophil‐Macrophage Transwell Co‐Culture

A Transwell system was utilized to evaluate indirect, neutrophil‐mediated macrophage polarization. Primary neutrophils (6 × 10^5^ cells) were seeded in the upper chamber and RAW264.7 cells (1.2 × 10^6^ cells) were seeded in the lower chamber, treated with non‐lethal concentrations of Zn alloy extracts (50%, 25%, and 12.5%). After 24 h of co‐culture, RNA from the lower‐chamber macrophages was extracted for RT‐PCR analysis. For each concentration, assays were performed with three replicates, and the entire experiment was repeated three times independently. For transcriptomic analysis (RNA‐seq), the 12.5% extract group was compared to a control group of untreated RAW264.7 cells. RNA was extracted and sequenced from three independent biological replicates per condition (*n* = 3, independent samples). Bioinformatic analyses were conducted using an online platform (http://www.bioinformatics.com.cn).

#### 5.5.6 Osteogenic Induction of MC3T3‐E1 Pre‐Osteoblasts

Collect the fresh supernatant following transwell culture. The supernatant underwent centrifugation for 5 min to remove residual cells, followed by filtration through a 0.22 µm filter, and subsequent storage at −80 °C for future use. This supernatant was then combined with fresh MEM‐α medium in a 1:2 ratio to produce conditioned medium. In the case of MC3T3‐E1 cells (*RRID: CVCL_5437*), conditioned medium containing osteogenic components (10 mM β‐glycerophosphate and 0.25 mM ascorbic acid) was employed to induce osteogenic differentiation. MC3T3‐E1 cells were seeded in 24‐well plates at a density of 1 × 10^4^ cells per well. Following 14 days of osteogenic induction in conditioned medium, alizarin red S (ARS) staining were performed to assess osteogenic differentiation. The conditioned medium was refreshed every other day.

### 5.6 Surgical Procedure

All animal surgeries were conducted in compliance with the ARRIVE guidelines. New Zealand white rabbits were purchased from Shanghai Jiagan Biotechnology Co., Ltd (Shanghai, China; *RRID: NCBITaxon_9986*). The experimental protocol received Ethical Approval (No. 2025.228) from the Animal Ethics Committee of Renji Hospital, Shanghai Jiao Tong University School of Medicine.

Implants composed of pure Zn and binary Zn alloys (Φ1.0 × 10 mm) were fabricated from ECAP‐processed rods. A male New Zealand white rabbit femoral implantation model was employed, and all surgical procedures were conducted under sterile conditions. Anesthesia was induced via intramuscular injection of ketamine (35 mg·kg^−^^1^) and 2% xylazine (5 mg·kg^−^^1^). Each rabbit was secured in a supine position with the right knee placed in a hyper‐flexed posture. The surgical site was shaved, depilated, and rigorously disinfected with povidone‐iodine and 75% ethanol. A cylindrical hole (1.2 mm in diameter) was drilled perpendicular to the long axis at the midshaft of the femur. Following irrigation of the bone cavity with sterile saline, the metal implant was inserted, and the surgical site was carefully sutured. Each experimental group was assigned six rabbits at a specified time point (one implant per animal). A total of 96 rabbits (12–16 weeks old, average weight 2.7 kg) were randomly assigned to experimental groups. Postoperatively, the animals were housed in a ventilated environment with free access to food and water. At 3 days, 14 days, 30 days and 90 days post‐implantation, the rabbits were euthanized, and the implanted femurs were harvested and fixed in 10% neutral buffered formalin for subsequent Micro‐CT and histological evaluation. Following extraction, the samples from each group were randomly assigned to micro‐CT analysis (*n* = 3, independent samples) or histology of decalcified sections (*n* = 3, independent samples). Detailed experimental methods and sample allocation were summarized in Table S4.

A femoral condyle fracture model was created in 20 rabbits (12‐16 weeks old). Following exposure of the femoral condyle via a lateral parapatellar approach, a longitudinal fracture was induced in the lateral condyle with a 1‐mm‐thick pendulum saw. The fracture was then stabilized with pure Ti or Zn‐0.8 Mg alloy screws placed perpendicular to the fracture line. Bone tissue samples were harvested 3 months post‐surgery for subsequent analysis. Body weight was measured before surgery and at each sampling point to ensure that any weight loss did not exceed 15% of the pre‐surgery, in accordance with animal welfare protocols (Figure S13).

### 5.7 Histological Preparation

Following saline rinsing, the samples were dehydrated through a graded ethanol series (75% to 100%), rendered transparent by immersion in xylene (2 × 8 min), and subsequently vacuum‐embedded in methyl methacrylate (MMA). In accordance with ISO 10993 guidelines, femoral shaft sections were prepared parallel to the long axis of the implant, taking the medullary canal position. The section thickness was precisely controlled at 50 ± 5 µm using a high‐precision grinding and polishing system (EXAKT 400CS). For decalcified bone specimens, the metallic implants were gently removed from the medullary cavity after initial tissue fixation and prior to paraffin embedding to allow for standard microtomy. This standardized procedure was applied to all experimental groups, including the pure Ti control. Subsequently, the femurs were immersed in ethylenediaminetetraacetic acid (EDTA) decalcifying solution for 6–8 weeks. After complete decalcification, the dehydrated samples were embedded in paraffin 5 µm longitudinal sections of femoral tissue for subsequent histological analysis.

### 5.8 Osteogenesis Analysis

Non‐decalcified bone sections were stained with methylene blue–acid fuchsin stains. High‐resolution whole‐slide images were subsequently acquired using a Pannoramic MIDI slide scanner (3DHISTECH) for osteogenesis analysis.

SHG imaging was conducted using a confocal fluorescence lifetime imaging microscope (FLIM, ISS, USA). SHG signals were generated at an excitation wavelength of 860 nm and detected at 420–430 nm (half the excitation wavelength). Images were acquired using a 10 × objective lens.

The implanted femurs were scanned using a high‐resolution micro‐CT system (SkyScan1176, Bruker, USA) at 90 days post‐implantation (*n* = 3 independent samples). Scans were performed with a voxel resolution of 18 µm under the parameters of 40 kV and 250 µA. The acquired data were reconstructed using Mimics Research software (Materialise, Belgium).

### 5.9 Histology, Immunohistochemistry and Immunofluorescence Staining

Specimens with a thickness of 5 µm were subjected to H&E staining, Masson's trichrome staining, and immunological evaluation. Inflammatory responses, tissue integration, and bone regeneration at defect sites were evaluated by H&E staining and Masson's trichrome staining. For immunohistochemical analysis, paraffin sections were incubated overnight at 4 °C with primary antibodies against Ly6g (1:200 dilution; Abcam, Cat# ab25377; *RRID: AB_470492*) and osteocalcin (OCN, 1:200 dilution; Proteintech, Cat# 23418‐1‐AP; *RRID: AB_2879275*), followed by incubation with horseradish peroxidase‐conjugated secondary antibodies for 1 h at room temperature in the dark. For immunofluorescence staining, sections were incubated overnight at 4 °C with primary antibodies against CD206 (1:200 dilution, Santa Cruz, Cat# sc‐58986; *RRID: AB_2144945*), TNF‐α (1:200 dilution; Proteintech, Cat# 60291‐1‐Ig; *RRID: AB_2833255*), TGF‐β1 (1:200 dilution; Proteintech, Cat# 81746‐2‐RR; *RRID: AB_3670503*) and a Zn^2+^‐sensitive fluorescent probe (FluoZin‐3, AM, 1:200 dilution), followed by incubation with Alexa Fluor 488/594/647‐labeled secondary antibodies. Nuclei were counterstained with DAPI, and washed thoroughly with PBS. Stained sections were imaged using a digital slide scanner (3DHISTECH, Hungary), and quantitative analysis was performed using Image‐Pro Plus 6.0 software (Media Cybernetics), *n* = 3, independent samples.

### 5.10 Cross Sectional Analysis

Hard tissue specimens were sectioned into slices approximately 1 mm thick. Cross‐sectional samples were then prepared by sequential grinding with 7000‐grit silicon carbide (SiC) paper, followed by final polishing using a 0.1 µm diamond suspension to obtain a smooth, artifact‐free surface. Sections were collected from the femoral shaft for subsequent microstructural evaluation. At least three sections were prepared for each experimental group to ensure data reliability. Prior to SEM analysis, all polished specimens were sputter‐coated with a thin layer of gold to enhance conductivity. Systematic macroscopic and microscopic observations were conducted on the implantation sites of all samples with BSE microscopy, and the interfacial composition between the implants and bone tissue was characterized and verified through elemental distribution scanning via EDS. For quantitative analysis of osteogenesis, a region of interest (ROI) with a diameter of 1.4 mm, encompassing the implants. Three independent samples per group were analyzed for in vivo quantitative of new bone formation.

### 5.11 Statistical Analysis

The data were presented as mean ± SD. All statistical analyses were conducted using GraphPad Prism 10.5.0 (GraphPad Software, San Diego, CA, USA) and OriginPro 2016 (Originlab, Northampton MA, USA). For in vitro assays (e.g., qPCR and RNA‐seq), statistical comparisons between two groups were performed using an unpaired two‐tailed Student's t‐test. For multiple group comparisons, one‐way analysis of variance (ANOVA) followed by Tukey's post‐hoc test was utilized. These parametric tests were applied as the relative expression data (log‐transformed ΔCt values) generally approximate a normal distribution with low technical variance. For in vivo quantitative evaluations, the biological sample size was ethically minimized to *n* = 3 per group in strict adherence to the 3Rs principle. Given that normality and equal variance assumptions cannot be robustly verified at this restricted sample size, non‐parametric tests. Specifically, the Kruskal‐Wallis test with Dunn's post hoc comparison was applied. Differences were considered statistically significant when *p < 0.05, **p < 0.01, ***p < 0.001, ****p < 0.0001. For comparisons where *p* > 0.05, we explicitly avoided over‐interpreting significance. Instead, these results were evaluated and described based on fold‐changes or biological trends.

## Conflicts of Interest

Yufeng Zheng is the inventor on a Chinese invention patent related to Zn‐Mg alloys (Patent No: ZL201410415524.7), which has been licensed for the development of orthopedic medical devices. The findings of this study establish a theoretical foundation and composition design rationale for biodegradable Zn alloys. This research may provide a supportive theoretical basis for future translational efforts, such as the clinical evaluation of Zn‐Mg alloy systems (e.g., ChiCTR2100051050), although the present study is not directly associated with the execution of that specific clinical trial. The remaining authors declare no conflict of interest.

## Supporting information




**Supporting File**: advs76084‐sup‐0001‐SuppMat.docx.

## Data Availability

The data that support the findings of this study are available from the corresponding author upon reasonable request.
