# Supplementary material for: Micro‐Galvanic Coupling Programs the Therapeutic Zinc Ion Window to Reconfigure Immune Cascades for Pro‐Regenerative Bone Healing
Source: Adv Sci (Weinh). 2026 Jun 12;13(50):e76084. doi: 10.1002/advs.76084 (PMC13336426; doi:10.1002/advs.76084)
Supplement: Supplementary file 1 [file ADVS-13-e76084-s001.docx]

Supporting Information

Micro-galvanic coupling programs the therapeutic zinc ion window to reconfigure immune cascades for pro-regenerative bone healing

*Chaoyang Sun, Bo Jia, Jiahui Shi, Shuang Li, Guo Bao, Dong Bian, Kai Chen, Junlong Tan, Xuenan Gu, Yan Guan, Yu Qin*, Xinhua Qu*, Xiaogang Wang*, Yufeng Zheng*, Hongtao Yang**

**The PDF file includes:**

Figs. S1 to S13

Tables S1 to S4

**Figure S1.** Microstructure characterization of Zn-0.8Mg and Zn-0.8Fe model alloys. **A.** Standard electrode potential differences between Zn and alloying elements. **B.** Solid solubility of alloying elements in Zn. **C.** Equal Channel Angular Pressing to obtain a Zn alloy with uniform dispersion of the second phase.

**Figure S2.** **A**. Surface potential characterization of pure Zn. **B**. Scanning vibrating electrode technique (SVET) mapping of pure Zn surface current density distribution after immersion in simulated body fluids (SBF) for 24 h and corrosion morphology after immersed in saline with pH = 4 for 7 days.

**Figure S3.** The effect of grain size on local galvanic corrosion was simulated by pure Zn and Mg_2_Zn_11_. **A.** The left side is the model diagram of the area ratio of cathode to anode. The right side is the electrode designed according to the model diagram, and the contact interface is bonded with conductive silver glue. **B.** A schematic diagram of a three-electrode system for polarization polarization (PDP) test. **C** Anode and cathode surface morphology before (left) and after (right) PDP test. **D.** Corrosion current density of electrodes with anode-cathode area ratios of 1:4 and 1:1.

**Figure S4.** **A.** HL-60 cells induced neutrophil staining. DAPI (bule) and free Zn^2+^ (green)) after co-culture with alloy extracts for 48 h. **B.** Cell viability of HL-60 cells induced neutrophils cultured for 48 h with 100% Zn alloy extracts (left, *n*=3, independent samples), and concentration of metal elements for Zn alloys extracts (right). P-values are calculated using one-way ANOVA with Tukey’s post hoc test, **p* < 0.05, ***p* < 0.01, ****p* < 0.001, *****p* < 0.0001.

**Figure S5.** Phenotypic changes of mouse primary bone marrow neutrophils cultured with different concentrations of pure zinc extract for 24 h. iNOS and IL-1β represent N1 type, while Arg1 and TGF-β represent N2 type (*n*=3, independent samples). Data were expressed as mean ± SD. P-values are calculated using one-way ANOVA with Tukey’s post hoc test, **p* < 0.05, ***p* < 0.01, ****p* < 0.001, *****p* < 0.0001.

**Figure S6.** The viability of RAW264.7 after co-culture with pure Zn extracts for 24 h (*n*=3, independent samples). Data were expressed as mean ± SD. P-values are calculated using one-way ANOVA with Tukey’s post hoc test, **p* < 0.05, ***p* < 0.01, ****p* < 0.001, *****p* < 0.0001.

**Figure S7.** Representative micrograph of alizarin red S staining of MC3T3-E1 pre-osteoblasts after 14 days of osteogenic induction with conditioned medium.

**Figure S8.** **A.** Volcano plot of RNA sequencing data illustrating differentially expressed genes between the 12.5% Zn-0.8Mg extract-treated group and the control group. Upregulated genes are shown in red and downregulated genes in blue. **B.** Gene Ontology (GO) enrichment analysis of the differentially expressed genes (*n* = 3, independent samples). P-values were calculated using two-sided t-tests without adjustment, **p* < 0.05, ***p* < 0.01, ****p* < 0.001, *****p* < 0.0001.

**Figure S9.** Immunohistochemical staining of osteogenesis-related markers (ALP and OCN) one month after implantation.

**Figure S10.** X-ray images of rabbits implanted with pure Ti and Zn-0.8Mg alloy screws. The first row is immediately post-surgery, and the second row is 90 days after implantation. The left and right columns represent the pure Ti group and the Zn-0.8Mg alloy group, respectively.

**Figure S11.** Immunohistochemical staining of OCN, OPG (osteogenic-related makers) after 3 months implantation of pure Ti and Zn-0.8Mg alloy screws in rabbit femoral. Positive areas are marked by asterisks.

**Figure S12.** Flow cytometric result of isolated neutrophils stained with CD11b (PE) and Ly6g (FITC).

**Figure S13**. *In vivo* systemic biosafety evaluation via longitudinal body weight monitoring. Serial measurements of body weight across all experimental groups over the 90-day postoperative period. Due to scheduled animal sacrifices at intermediate experimental time points, the total number of assessed animals (*n*) was as follows: pre-surgery and day 3 (*n* = 116), day 14 (*n* = 92), day 30 (*n* = 68), and day 90 (*n* = 44). The red dashed line denotes the critical endpoint threshold, set at a 15% decline from the initial average weight.

**Table S1.** The fitted results of elements in equivalent circuits of EIS

| Sample | *Rs*  （Ω） | *R_P_*  （Ω） | *E_Corr_*  (V vs. SHE) | *icorr*  (μA/cm^2^) |
| --- | --- | --- | --- | --- |
| Zn | 75.531 | 1667.8 | -1.183 | 1.368 |
| Zn-0.8Mg | 64.545 | 6437.7 | -1.173 | 0.911 |
| Zn-0.8Fe | 83.23 | 1642.1 | -1.165 | 2.256 |

**Table S2.** Chemical composition of pure Zn and binary Zn alloys

| Materials | Nominal Composition (wt. %) | | Actual Composition (wt. %) | | |
| --- | --- | --- | --- | --- | --- |
|  | Alloy contents | Zn | Alloy contents | | Zn |
| Pure Zn | - | 99.99 | - | 99.99 | |
| Zn-0.8Mg | 0.8 | Bal. | 0.778 | Bal. | |
| Zn-0.8Fe | 0.8 | Bal. | 0.691 | Bal. | |

**Table S3.** Primer sequences of migration-related genes for RT-PCR

| Primer | Sequences |
| --- | --- |
| Gapdh | AGGTCGGTGTGAACGGATTTG  TGTAGACCATGTAGTTGAGGTCA |
| TNF-α | CCCTCACACTCAGATCATCTTCT  GCTACGACGTGGGCTACAG |
| IL-10 | GCTCTTACTGACTGGCATGAG  CGCAGCTCTAGGAGCATGTG |
| iNOS | GTTCTCAGCCCAACAATACAAGA  GTGGACGGGTCGATGTCAC |
| TGF-β1 | CTTCAATACGTCAGACATTCGGG  GTAACGCCAGGAATTGTTGCTA |
| Arg1 | CTCCAAGCCAAAGTCCTTAGAG  AGGAGCTGTCATTAGGGACATC |
| Il-1β | GCAACTGTTCCTGAACTCAACT  ATCTTTTGGGGTCCGTCAACT |
|  |  |

**Table S4.** Summary of *in vivo* experimental allocation and downstream analysis

| Experimental Group | Time point | Animals allocation (Biological *n)* | Sample allocation (Biological *n*) | Downstream Analyses |
| --- | --- | --- | --- | --- |
| Pure Ti | Day 3 | 6 | Decalcified, *n* = 3 | Immunofluorescence, Immunohistochemistry, HE and Masson staining |
|  |  |  | Undecalcified, *n* = 3 | Methylene blue-acid fuchsin staining, SEM, |
|  | Day 14 | 6 | Decalcified, *n* = 3 | Immunofluorescence, HE and Masson staining |
|  |  |  | Undecalcified, *n* = 3 | Methylene blue-acid fuchsin staining, SEM, |
|  | Day 30 | 6 | Decalcified, *n* = 3 | Immunohistochemistry, HE and Masson staining |
|  |  |  | Undecalcified, *n* = 3 | Methylene blue-acid fuchsin staining, SEM, |
|  | Day 90 | 6 | Decalcified, *n* = 3 | HE and Masson staining |
|  |  |  | Undecalcified, *n* = 3 | CT, Methylene blue-acid fuchsin staining, SEM, TEM |

*Note*: The methodology for *in vivo* experimental allocation and downstream analysis was consistent across all experimental groups. This table presents the detailed design using the pure Ti group as a representative example.
